# MARCH6 suppresses Tembusu virus replication by targeting viral NS5 protein for TOLLIP-mediated selective autophagic degradation

**DOI:** 10.1128/jvi.00735-25

**Published:** 2025-06-13

**Authors:** Peng Zhou, Wanrong Wu, Jiani Wei, Yueshan Yang, Anan Jongkaewwattana, Yuncai Xiao, Hui Jin, Hongbo Zhou, Rui Luo

**Affiliations:** 1 State Key Laboratory of Agricultural Microbiology, College of Veterinary Medicine, Huazhong Agriculture University 124443 , Wuhan, China; 2 Key Laboratory of Preventive Veterinary Medicine in Hubei Province, The Cooperative Innovation Center for Sustainable Pig Production, Wuhan, China; 3 Virology and Cell Technology Research Team, National Center for Genetic Engineering and Biotechnology (BIOTEC), National Science and Technology Development Agency (NSTDA) 61191, Pathum Thani, Thailand; University of Kentucky College of Medicine, Lexington, Kentucky, USA

**Keywords:** MARCH6, TMUV replication, NS5, TOLLIP, autophagic degradation

## Abstract

The E3 ligase membrane-associated RING finger 6 (MARCH6) plays a pivotal role in various cellular processes; however, its role in viral defense remains largely unexplored. In this study, we have elucidated a novel antiviral mechanism of avian MARCH6 against duck Tembusu virus (TMUV), revealing a previously uncharacterized host defense strategy. Notably, MARCH6 expression was significantly upregulated during TMUV infection in several duck cell lines, suggesting a conserved cellular response. Functional analyses revealed that overexpression of MARCH6 effectively suppressed TMUV replication, whereas its knockdown markedly enhanced viral replication. Mechanistically, MARCH6 directly interacts with the viral non-structural protein 5 (NS5), mediating its targeted degradation through an unprecedented E3 ligase activity-independent mechanism. Moreover, MARCH6 recruits the autophagic cargo receptor TOLLIP, which facilitates the NS5-TOLLIP interaction independent of ubiquitin signaling and subsequently directs NS5 to phagophores for degradation. These findings reveal a novel antiviral mechanism that focuses on the MARCH6-NS5-TOLLIP axis and represents a critical host defense strategy against viral infections. This study not only provides insights into the antiviral functions of MARCH6 but also emphasizes the importance of selective autophagy as a fundamental mechanism to control viral infection.

**IMPORTANCE:**

TMUV, an emerging pathogenic flavivirus, has rapidly spread across major duck farming regions in Asia since 2010, causing substantial economic losses in the duck industry. More recently, TMUV has expanded its host range, raising concerns about its potential threat to mammals. Understanding TMUV-host interactions is essential for developing effective treatments and vaccines. Here, we uncover a previously uncharacterized role of avian MARCH6 in antiviral defense against TMUV. We demonstrate that MARCH6 restricts TMUV replication through an E3 ligase activity-independent mechanism by targeting the viral NS5 protein for degradation. Notably, MARCH6 promotes NS5 degradation via selective autophagy by recruiting the cargo receptor TOLLIP, bypassing conventional ubiquitin signaling. These findings reveal a novel host antiviral strategy centered on the MARCH6-NS5-TOLLIP axis, broadening our understanding of selective autophagy in antiviral defense.

## INTRODUCTION

Tembusu virus (TMUV), a member of the *Orthoflavivirus* genus within the *Flaviviridae* family, was first isolated from *Culex tritaeniorhynchus* mosquitoes in Malaysia in 1955 (1, 2). In 2010, TMUV triggered large-scale outbreaks in China, rapidly disseminating to most duck-producing regions of Malaysia, Thailand, and other Southeast Asian countries (3–5). Infected ducks exhibited severe egg-drop syndrome, stunted growth, and neurological symptoms, resulting in substantial economic losses to the duck industry (6, 7). The TMUV genome comprises a positive-sense, single-stranded RNA of approximately 11 kb, encoding a single polyprotein (8). This polyprotein is cleaved by viral and host proteases into three structural proteins (C, prM, and E) and seven non-structural proteins (NS1, NS2A, NS2B, NS3, NS4A, NS4B, and NS5) (9). The structural proteins primarily facilitate viral attachment, membrane fusion, and virion assembly, while the non-structural proteins play critical roles in viral replication and modulation of the host’s immune responses (10–12). Among these, NS5 is the largest protein and contains two essential enzymatic domains: the N-terminal methyltransferase (MTase) domain and the C-terminal RNA-dependent RNA polymerase (RdRp) domain (13). These domains are essential for viral RNA synthesis, capping, and evasion of the host immune response (14–16).

Autophagy is a highly conserved intracellular degradation process characterized by the formation of double-membrane autophagosomes that fuse with lysosomes to degrade and recycle cellular components, including damaged organelles, misfolded or aggregated proteins, and invading pathogens (17–19). Although autophagy was initially regarded as a non-selective bulk degradation process, various forms of selective autophagy have now been well characterized (20–23). In selective autophagy, specific substrates are recognized by a diverse array of cargo receptors, including sequestosome 1 (SQSTM1/p62), optineurin (OPTN), NBR1 autophagy cargo receptor (NBR1), toll interacting protein (TOLLIP), calcium binding and coiled-coil domain-containing protein 2 (CALCOCO2/NDP52), and BCL2 interacting protein 3 like (BNIP3L/NIX) (24, 25). Following substrate recognition, these receptors bind directly to Atg8 family proteins on phagophores via their MAP1LC3/LC3-interacting region, thereby facilitating the transport of cargo to lysosomes for degradation (26, 27). Beyond maintaining cellular homeostasis, selective autophagy plays a crucial role in host antiviral defense by targeting and degrading viral components (28, 29). For example, the cargo receptor TOLLIP recognizes the NS3 protein of dengue virus (DENV) after it is ubiquitinated by the E3 ubiquitin ligase cullin 2 and promotes its autophagic degradation, thereby inhibiting DENV infection (30). Similarly, SQSTM1/p62 inhibits Zika virus replication by binding to NS3 and NS5, directing them toward autophagic degradation (31). However, the role of selective autophagy in regulating TMUV replication remains poorly understood.

The membrane-associated RING-CH (MARCH) family of proteins represents a subfamily of E3 ubiquitin ligases characterized by a highly conserved N-terminal RING-CH domain and structurally diverse C-terminal transmembrane (TM) domains (32–34). The RING-CH domain is essential for the E3 ubiquitin ligase activity of MARCH proteins, which facilitates their ability to modulate the stability, trafficking, and activity of various cellular proteins through ubiquitination (35–38). Beyond their role in cellular processes, MARCH proteins have emerged as key antiviral factors that mediate the degradation or intracellular sequestration of viral components. For example, MARCH8 restricts influenza A virus infection by interacting with the M2 protein, triggering its K63-linked polyubiquitination, and diverting it from the plasma membrane to lysosomes for degradation (39). Similarly, mammalian MARCH1 and MARCH2 diminish HIV-1 infectivity by impairing the translocation of envelope glycoproteins to the cell surface, thereby reducing their incorporation into virions (40). However, the antiviral functions of MARCH6 remain poorly characterized. In this study, we identified avian MARCH6 as a novel host restriction factor against TMUV infection. Mechanistically, MARCH6 is upregulated during TMUV infection and facilitates the interaction between viral NS5 and the cargo receptor TOLLIP, thereby promoting the degradation of NS5 via TOLLIP-mediated selective autophagy. Our findings reveal a novel antiviral mechanism of MARCH6, wherein it suppresses flavivirus infection through the selective autophagy pathway.

## RESULTS

### TMUV infection induces MARCH6 expression

**Fig 1:**
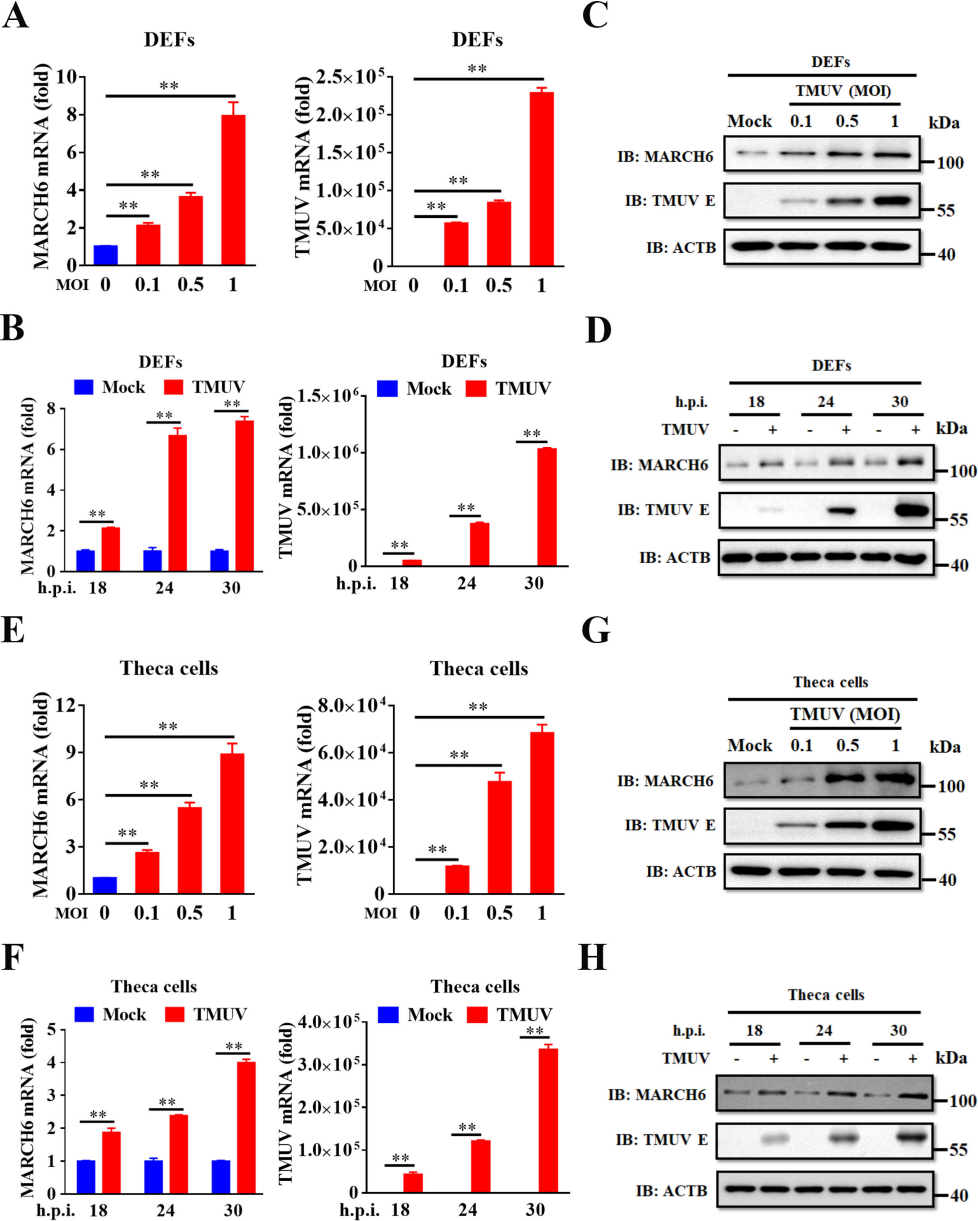
TMUV infection induces MARCH6 expression. (**A, C, E, and G**) DEFs (**A and C**) or duck theca cells (**E and G**) were infected with TMUV at multiplicities of infection (MOIs) of 0.1, 0.5, and 1.0 for 24 h. The levels of MARCH6 mRNA and viral RNA were measured by RT-qPCR (**A and E**), and endogenous MARCH6 and viral E proteins were detected by Western blotting (**C and G**). (**B, D, F, and H**) DEFs (**B and D**) or duck theca cells (**F and H**) were infected with TMUV at an MOI of 0.1, and cells were harvested at 18, 24, and 30 h post-infection (h.p.i.), respectively. The levels of MARCH6 mRNA and viral RNA were quantified by RT-qPCR (**B and F**), and endogenous MARCH6 and viral E proteins were analyzed by Western blotting (**D and H**). (A, B, E, and F) Data are expressed as mean ± standard error of the mean of three independent experiments (Student’s *t*-test; **, *P* < 0.01).

To address the unresolved role of MARCH6 in the antiviral response, we sought to investigate its functional role during viral infection. Initially, we investigated whether TMUV infection modulates MARCH6 expression. To this end, duck embryo fibroblasts (DEFs) were infected with the TMUV strain MC, and MARCH6 transcript and protein levels were quantified using real-time quantitative PCR (RT-qPCR) and Western blotting. Quantitative RT-qPCR and immunoblot analyses revealed a progressive, time- and dose-dependent increase in both duck MARCH6 mRNA and protein levels during TMUV infection (Fig. 1A through D). Parallel assessments of viral replication kinetics, confirmed by measuring viral E protein and RNA levels, correlated with MARCH6 induction (Fig. 1A through D). To evaluate cell-type specificity, we infected theca cells isolated from duck ovarian follicles with TMUV and observed comparable upregulation of duck MARCH6 mRNA and protein (Fig. 1E through H). Collectively, these results indicate that TMUV infection robustly induces MARCH6 expression in different avian cell types, suggesting a possible functional role in host antiviral response.

### MARCH6 restricts TMUV replication through an E3 ligase-independent mechanism

**Fig 2:**
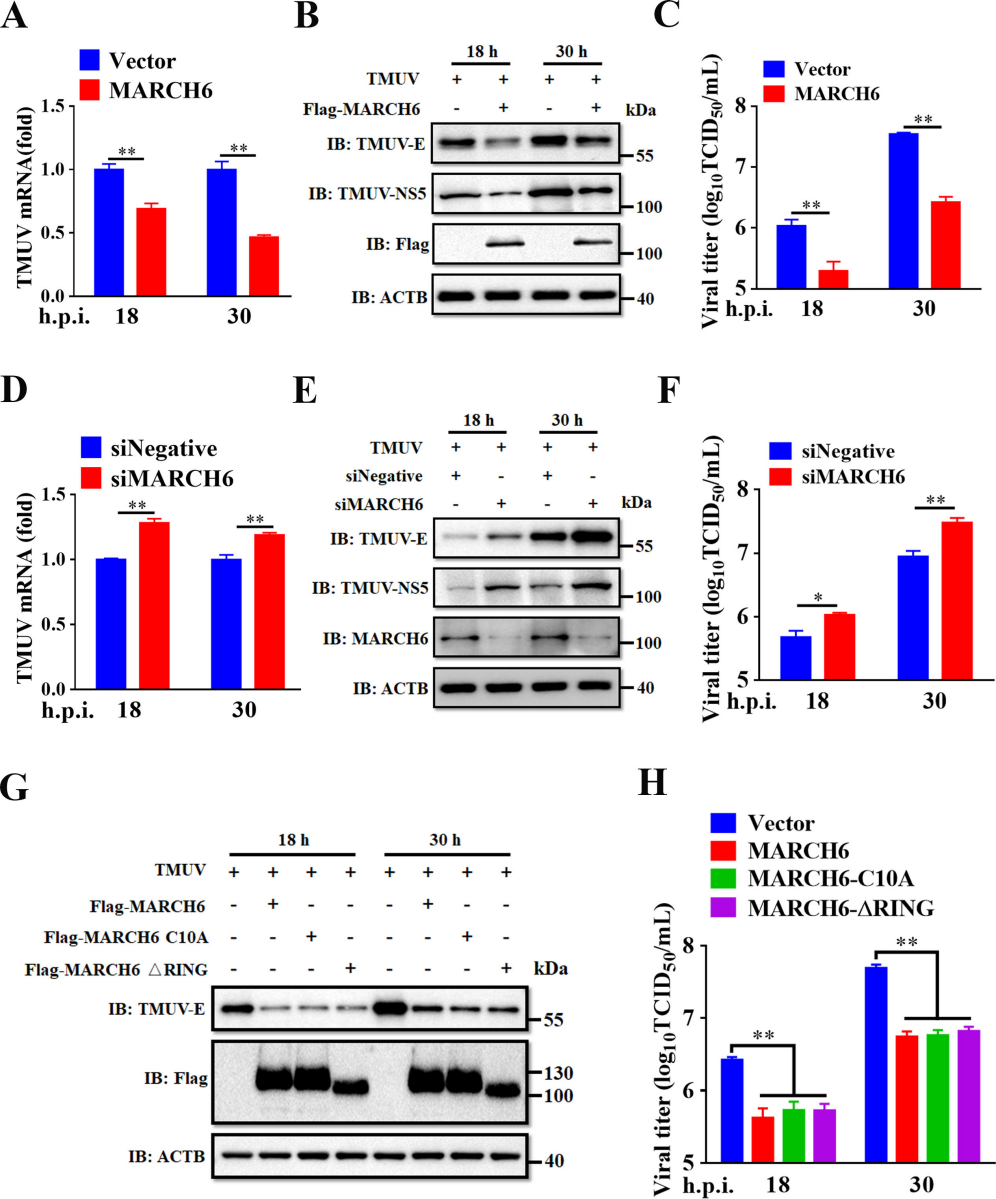
MARCH6 restricts TMUV replication through an E3 ligase-independent mechanism. (**A–C**) DEFs were transfected with plasmids encoding Flag-MARCH6 or empty vector for 24 h, followed by infection with TMUV at an MOI of 0.1. Viral mRNA (**A**) and E/NS5 protein (**B**) levels were analyzed by RT-qPCR and Western blotting at 18 and 30 h.p.i. Viral titers in culture supernatants (**C**) were quantified using the TCID_50_ assay. (**D–F**) DEFs were transfected with either siMARCH6 or a negative control siRNA (siNegative) for 24 h, followed by TMUV infection with an MOI of 0.1. The levels of viral mRNA (**D**) and E/NS5 protein (**E**) were determined at 18 and 30 h.p.i. by RT-qPCR and Western blotting, respectively. Viral titers in culture supernatants (**F**) were determined by TCID_50_ assay. (**G and H**) DEFs were transfected with plasmids encoding MARCH6 or its mutants for 24 h, followed by TMUV infection with an MOI of 0.1. E protein expression was determined at 18 and 30 h.p.i. using Western blotting (**G**), and viral titers in culture supernatants were quantified using the TCID_50_ assay (**H**). (A, C, D, F, and H) Data are presented as mean ± standard error of the mean of three independent experiments (Student’s *t*-test; *, *P* < 0.05; **, *P* < 0.01).

To investigate the effect of MARCH6 on TMUV replication, DEFs were transfected with Flag-tagged MARCH6 expression plasmids or an empty vector control, followed by TMUV infection. RT-qPCR showed a time-dependent reduction of viral genomic RNA levels in MARCH6-expressing cells, with 1.4-fold at 18 h post-infection (h.p.i.) and 2.2-fold at 30 h.p.i. decrease compared to vector controls (Fig. 2A). This antiviral effect was confirmed by reduced TMUV replication as evidenced by reduced viral E and NS5 protein expression (Fig. 2B) and lower infectious viral titers (Fig. 2C), quantified by immunoblotting and TCID_50_ assays. To further validate this observation, we synthesized three MARCH6-targeted small interfering RNAs (siRNAs); siMARCH6-3 achieved the highest knockdown efficiency (Fig. S1) and was selected for further experiments. Knockdown of MARCH6 resulted in a significant increase in viral RNA accumulation, synthesis of E and NS5 proteins, and production of infectious particles compared to the negative control siRNAs (siNegative) (Fig. 2D through F). Taken together, these data demonstrate that MARCH6 effectively suppresses TMUV replication.

Considering that MARCH6 belongs to the E3 ubiquitin ligase family and possesses a canonical RING-CH domain (41), we next investigated whether its enzymatic activity mediates antiviral function. To this end, we generated two loss-of-function mutants: a mutant with truncated RING domain (ΔRING) and a catalytically inactive mutant (C10A) in which the cysteine of the active site at position 10 was replaced by alanine. Unexpectedly, both mutants retained antiviral activity comparable to that of wild-type MARCH6, as shown by the equivalent reduction in E protein abundance (Fig. 2G) and viral titers (Fig. 2H). These results indicate that MARCH6 restricts TMUV replication independently of its E3 ubiquitin ligase activity.

### MARCH6 promotes the degradation of TMUV NS5 to suppress viral replication

**Fig 3:**
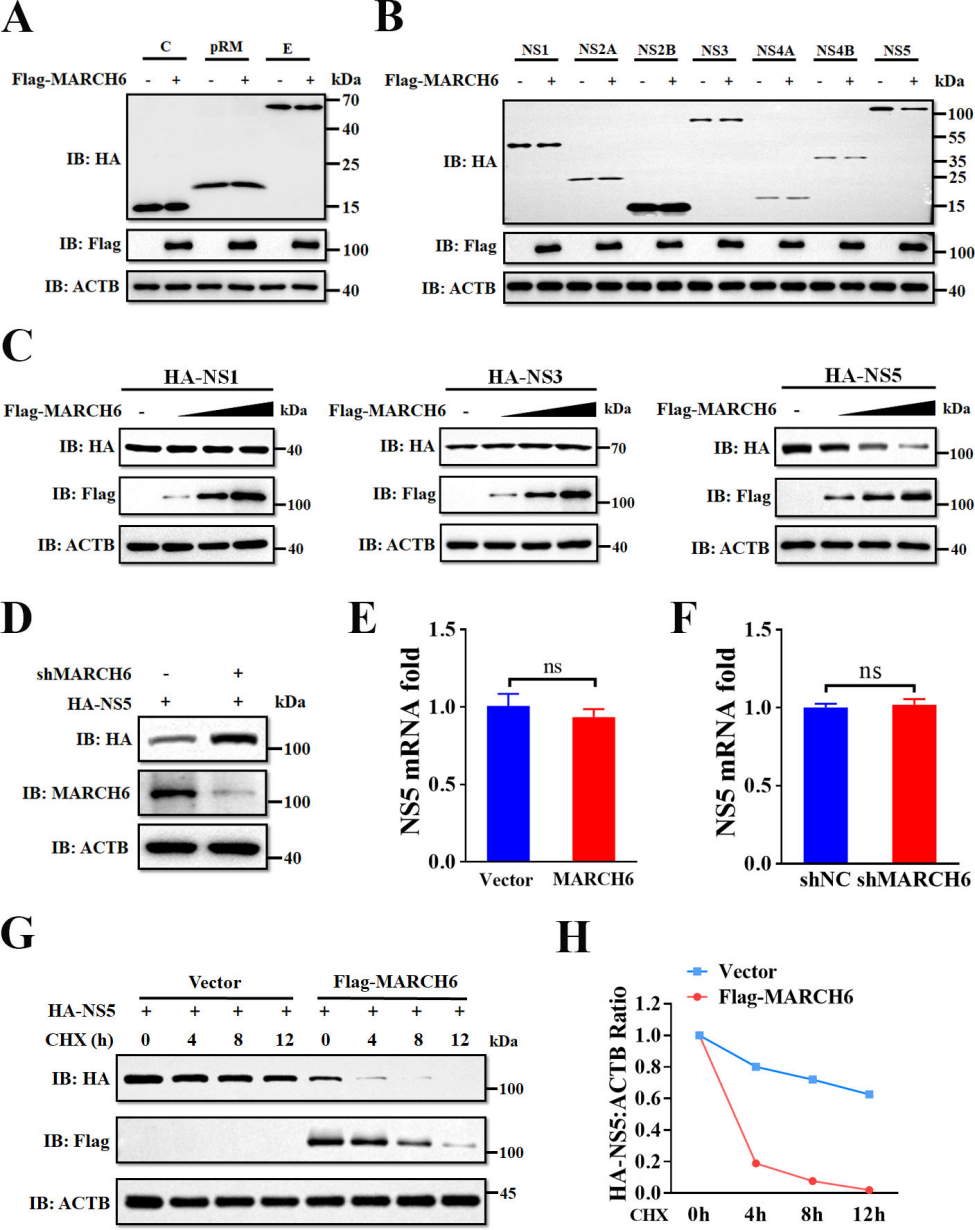
MARCH6 promotes the degradation of TMUV NS5 to suppress viral replication. (**A and B**) Immunoblot analysis of protein extracts from DEFs transfected with plasmids encoding Flag-MARCH6 together with TMUV structural proteins (**A**) or non-structural proteins (**B**). (**C**) Immunoblot analysis of protein extracts from DEFs transfected with plasmids encoding HA-tagged NS1, NS3, or NS5, along with increasing amounts of Flag-MARCH6. (**D**) Immunoblot analysis of protein extracts from DEFs transfected with plasmids encoding HA-NS5 and shMARCH6. (**E and F**) RT-qPCR analysis of NS5 mRNA levels in DEFs transfected with plasmids encoding HA-NS5 and either Flag-MARCH6 (**E**) or shMARCH6 (**F**). (**G**) Immunoblot analysis of protein extracts from DEFs transfected with plasmids encoding HA-NS5 and either Flag-MARCH6 or an empty vector for 24 h, followed by treatment with cycloheximide (CHX, 100 µg/mL) for the indicated times. (**H**) Quantification of NS5 protein levels from panel G, normalized to ACTB. (E and F) Data are presented as mean ± standard error of the mean of three independent experiments (Student’s *t*-test). ns, not significant.

To elucidate how MARCH6 inhibits TMUV replication, we co-transfected DEFs with Flag-MARCH6 and individual TMUV gene expression plasmids to examine the effect on viral protein expression. Western blotting analysis revealed that overexpression of MARCH6 specifically reduced NS5 protein levels (Fig. 3A and B), while no significant changes were observed for other viral proteins. To validate these results, we co-transfected DEFs with expression plasmids for NS1, NS3, or NS5 together with increasing amounts of MARCH6. As shown in Fig. 3C, NS5 expression decreased in a dose-dependent manner with increasing amounts of MARCH6, while NS1 and NS3 expression remained unchanged. Conversely, silencing MARCH6 using shRNA led to a significant increase in NS5 protein levels (Fig. 3D). Notably, neither manipulation altered NS5 mRNA levels (Fig. 3E and F), ruling out transcriptional regulation. Moreover, cycloheximide (CHX) chase experiments confirmed MARCH6 accelerated NS5 protein degradation (Fig. 3G and H), suggesting post-translational regulation. Together, these results demonstrate that MARCH6 selectively targets NS5 for degradation to restrict TMUV replication.

### MARCH6 directly binds TMUV NS5 via its transmembrane domain to promote degradation

**Fig 4:**
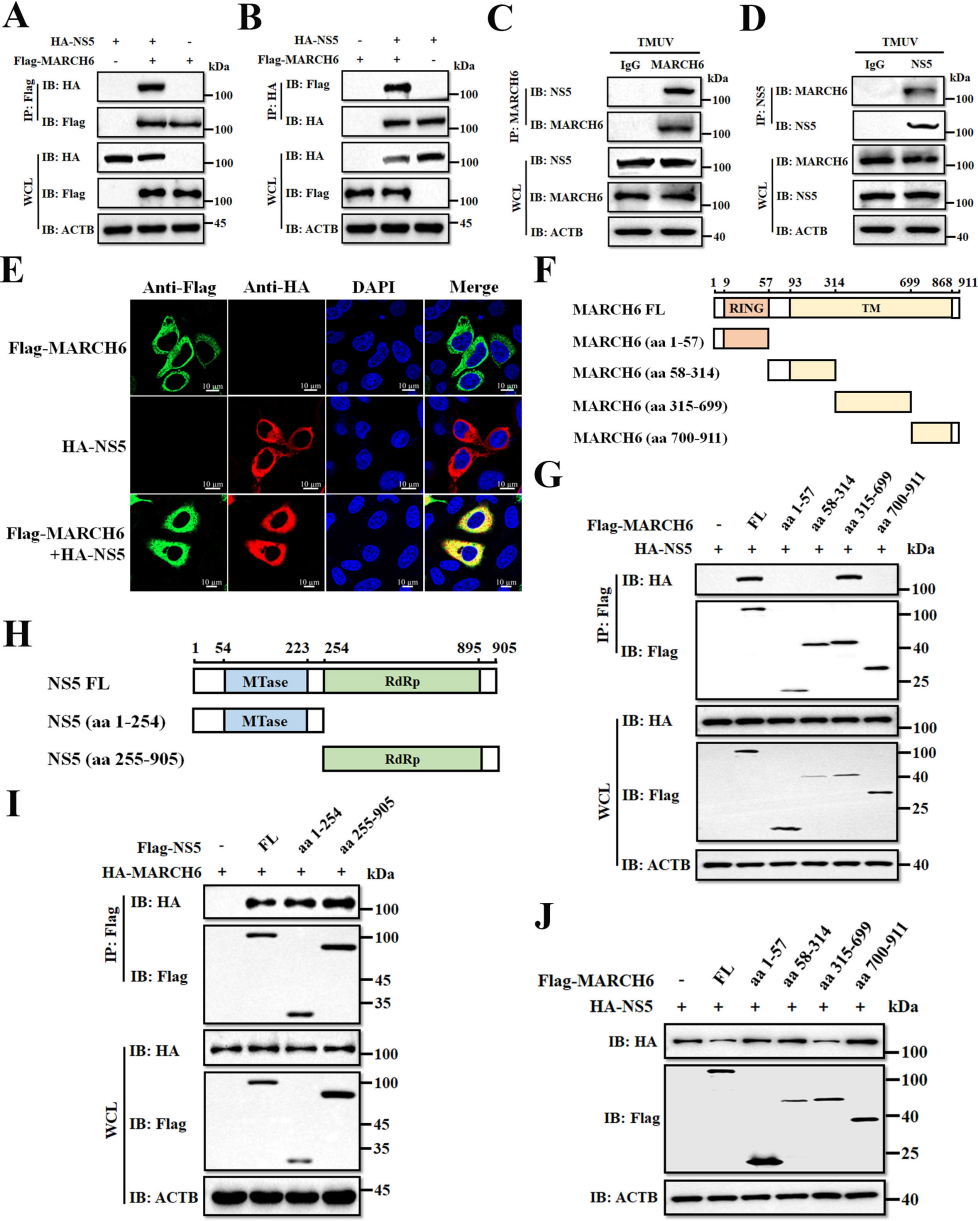
MARCH6 directly binds TMUV NS5 via its transmembrane domain to promote degradation. (**A and B**) Lysates from HEK-293T cells co-transfected with HA-NS5 and Flag-MARCH6 were subjected to co-IP with anti-Flag (**A**) and anti-HA (**B**) antibodies, followed by Western blotting analysis. (**C and D**) Lysates from DEFs infected with TMUV (MOI = 0.1) for 30 h were subjected to co-IP using anti-MARCH6 (**C**) and anti-NS5 (**D**) antibodies, followed by Western blotting analysis. (**E**) Co-localization of MARCH6 and NS5 proteins in HeLa cells expressing Flag-MARCH6 and HA-NS5 for 24 h. Nuclei were stained with 4′,6-diamidino-2-phenylindole (DAPI). Scale bars: 10 µm. (**F**) Schematic representation of MARCH6 domains. (**G**) Co-IP and immunoblotting analysis of lysates from HEK-293T cells transfected with Flag-MARCH6 and its truncated mutants, along with HA-NS5. (**H**) Schematic representation of TMUV NS5 domains. (**I**) Co-IP and immunoblotting analysis of lysates from HEK-293T cells transfected with Flag-NS5 or its truncated mutants, along with HA-MARCH6. (**J**) Immunoblot analysis of protein extracts from DEFs transfected with plasmids encoding Flag-MARCH6 or its deletion mutants, along with HA-NS5.

Given the role of MARCH6 in NS5 degradation, we next investigated whether MARCH6 physically interacts with NS5. Co-transfection of Flag-MARCH6 and HA-NS5 plasmids into HEK-293T cells, followed by immunoprecipitation with an anti-Flag antibody, revealed a specific interaction between MARCH6 and NS5 (Fig. 4A). Reciprocal co-immunoprecipitation (co-IP) experiments further confirmed this interaction (Fig. 4B). To determine whether endogenous MARCH6 interacts with native NS5 during TMUV infection, DEFs were infected with TMUV, and co-IP experiments were performed. Endogenous co-IP in TMUV-infected DEFs revealed native NS5 binding to MARCH6 during viral infection (Fig. 4C and D), while confocal microscopy showed cytoplasmic co-localization (Fig. 4E), consistent with the co-IP results. To investigate the mechanistic basis of this interaction, we generated a series of MARCH6 truncation mutants spanning amino acids (aa) 1–57, 58–314, 315–699, and 700–911 for co-IP assays (Fig. 4F). As shown in Fig. 4G, NS5 was found to interact specifically with the MARCH6 fragment comprising aa 315–699. To further identify the region of NS5 responsible for the interaction with MARCH6, we generated truncated NS5 constructs containing the MTase domain (aa 1–254) and the RdRp domain (aa 255–905) for co-IP experiments with MARCH6 (Fig. 4H). Interestingly, MARCH6 was found to interact with both the MTase and RdRp domains of NS5 (Fig. 4I). Since the MARCH6 fragment spanning amino acids 315–699 mediates interaction with NS5 (Fig. 4G), we further examined whether this region contributes to NS5 degradation. As expected, this fragment played a prominent role in promoting NS5 degradation (Fig. 4J). This observation is consistent with previous results showing that deletion of the RING domain of MARCH6 has no effect on the suppression of TMUV infection (Fig. 2G and H). Together, these results demonstrate that the TM domain of MARCH6 mediates a physical interaction with TMUV NS5, creating a structural platform for targeted degradation.

### MARCH6 hijacks autophagic machinery to degrade TMUV NS5

**Fig 5:**
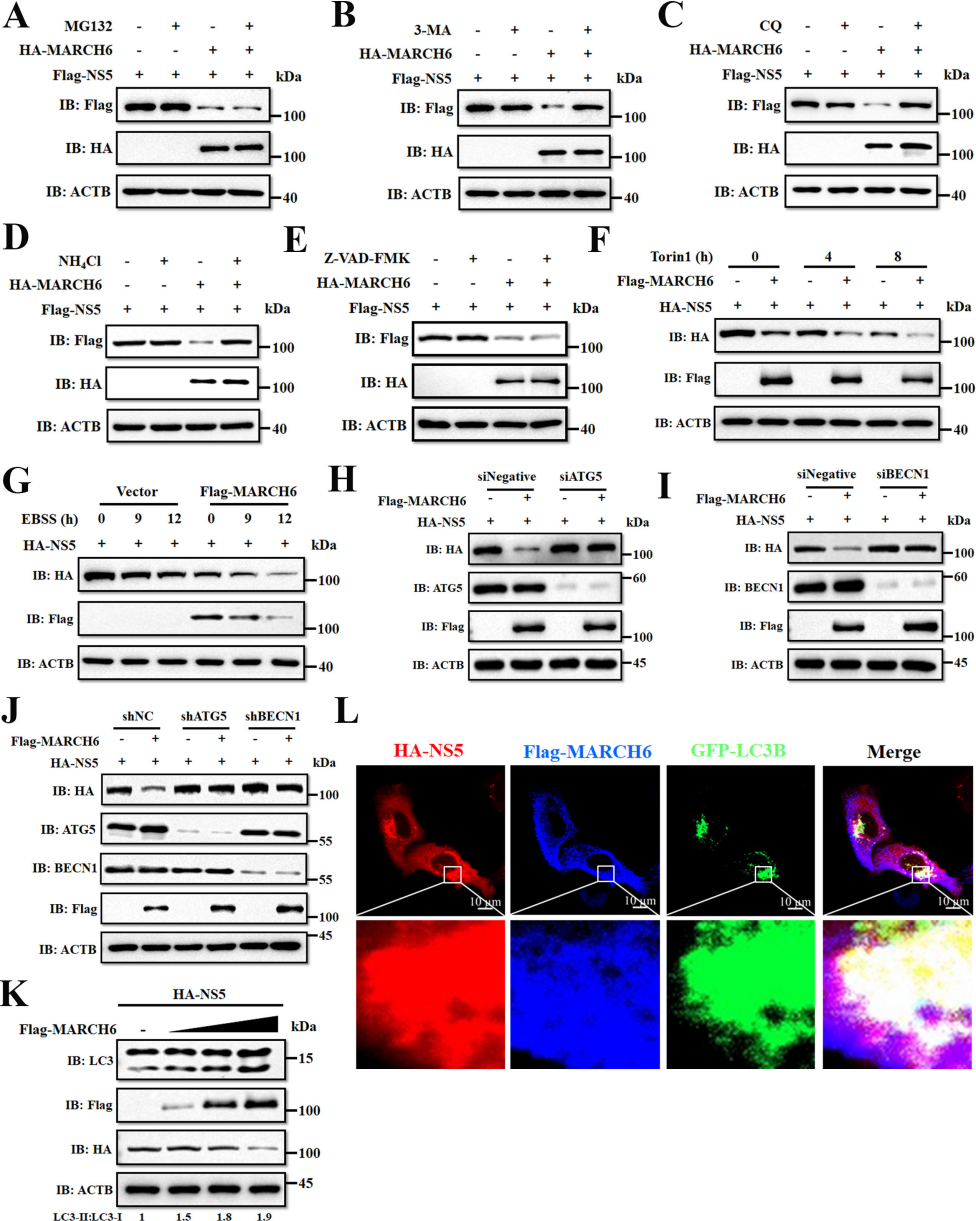
MARCH6 hijacks autophagic machinery to degrade TMUV NS5. (**A–E**) Immunoblotting analysis of DEFs co-transfected with HA-MARCH6 and Flag-NS5 for 24 h, followed by treatment with MG132 (10 µM) (**A**), 3-MA (10 mM) (**B**), CQ (10 µM) (**C**), NH_4_Cl (20 mM) (**D**), or Z-VAD-FMK (50 µM) (**E**) for 6 h. (**F**) Immunoblotting analysis of lysates from DEFs co-transfected with HA-NS5 and either Flag-MARCH6 or empty vector for 24 h, followed by treatment with Torin1 (250 nM) for the indicated times. (**G**) Immunoblot analysis of lysates from DEFs co-transfected with HA-NS5 and either Flag-MARCH6 or an empty vector for 24 h, followed by treatment with EBSS for the indicated times. (**H and I**) Immunoblot analysis of protein extracts from DEFs co-transfected with Flag-MARCH6 and HA-NS5, along with siATG5 (**H**) or siBECN1 (**I**) for 24 h. (**J**) Immunoblot analysis of protein extracts from DEFs co-transfected with Flag-MARCH6 and HA-NS5, together with shATG5 or shBECN1 for 24 h. (**K**) Immunoblot analysis of protein extracts from DEFs transfected with HA-NS5, along with either an empty vector or increasing amounts of plasmid expressing Flag-MARCH6. (**L**) HeLa cells co-transfected with Flag-MARCH6, GFP-LC3B, and HA-NS5 for 24 h were fixed and analyzed by immunofluorescence with anti-Flag and anti-HA antibodies and visualized by confocal microscopy. Scale bar: 10 µm.

Degradation of eukaryotic proteins occurs via three major pathways: apoptosis, ubiquitin-proteasome, and autophagy-lysosome systems (42). To identify the pathway of MARCH6-mediated NS5 degradation, we treated DEFs co-expressing Flag-NS5 and HA-MARCH6 with pathway-specific inhibitors, including the apoptosis inhibitor Z-VAD-FMK, the proteasome inhibitor MG132, and the autophagy inhibitors 3-methyladenine (3-MA), chloroquine (CQ), and NH_4_Cl. Strikingly, three distinct autophagy inhibitors (3-MA, CQ, and NH_4_Cl) reversed NS5 degradation, while apoptosis (Z-VAD-FMK) and proteasome inhibitors (MG132) showed no effect (Fig. 5A through E). This establishes autophagy as the primary degradation mechanism. Next, we induced autophagy with the mTOR inhibitor Torin1 or starvation under Earle’s Balanced Salt Solution (EBSS) culture conditions and found that both treatments synergistically enhanced MARCH6’s degradation (Fig. 5F and G). Moreover, knockdown of autophagy regulators ATG5 or BECN1 abrogated MARCH6-mediated NS5 reduction (Fig. 5H through J). Additionally, we also examined the impact of MARCH6 on autophagy levels in cells expressing NS5. Co-expression of Flag-MARCH6 and HA-NS5 in DEFs resulted in a dose-dependent increase in LC3-I to LC3-II conversion, indicating that MARCH6 may elevate autophagy levels in cells expressing NS5 (Fig. 5K). Confocal microscopy also revealed a co-localization of MARCH6 and NS5 on the LC3B-marked autophagosomes (Fig. 5L), further supporting the involvement of the autophagy pathway. Collectively, these results demonstrate that MARCH6 promotes the autophagic degradation of NS5.

### MARCH6 recruits TOLLIP as an autophagy cargo receptor for NS5 degradation

**Fig 6:**
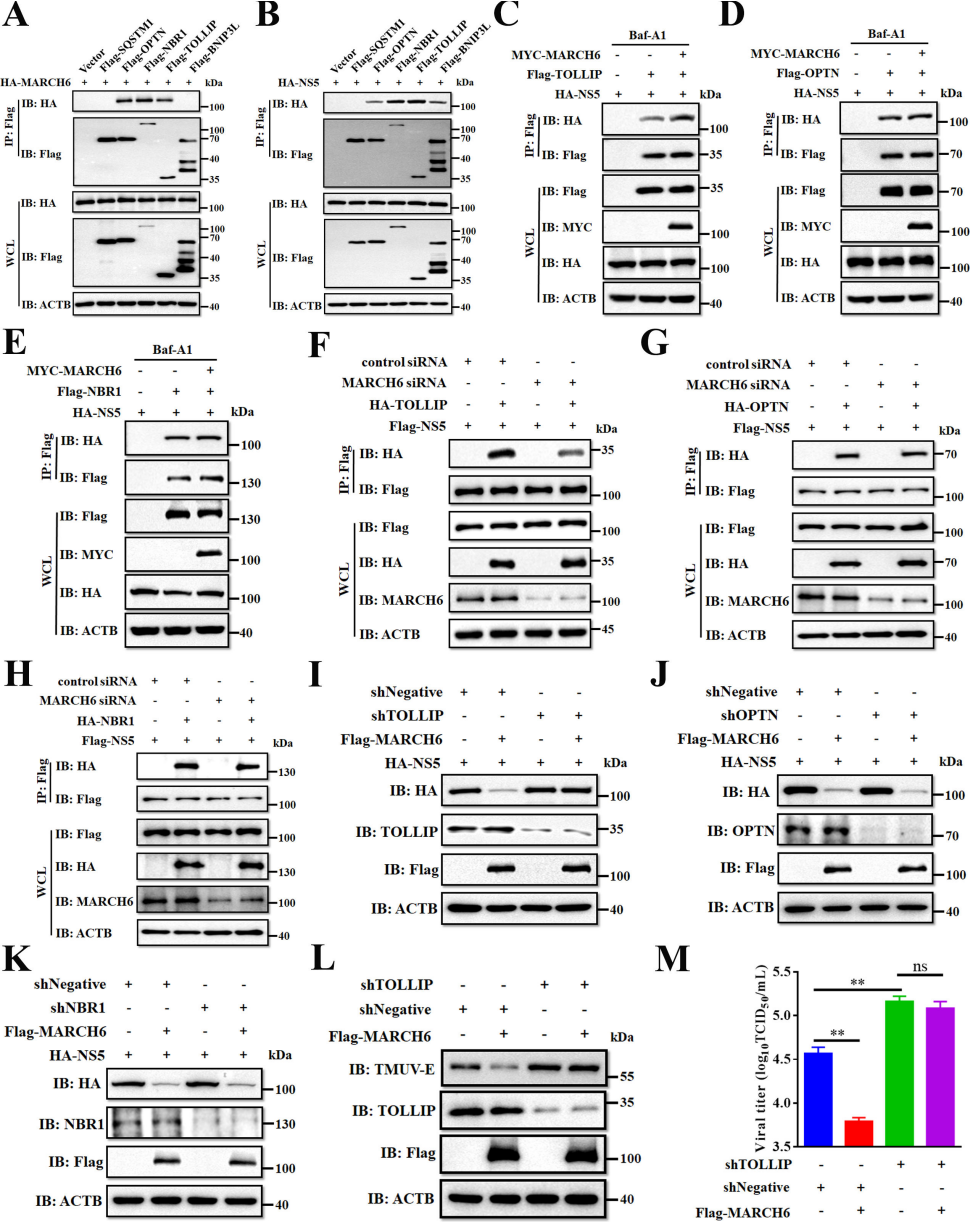
MARCH6 recruits TOLLIP as an autophagy cargo receptor for NS5 degradation. (**A and B**) Lysates of DEFs co-transfected with the indicated Flag-tagged cargo receptors and HA-MARCH6 (**A**) or HA-NS5 (**B**) were subjected to co-IP assays with an anti-Flag antibody, followed by Western blotting analysis. (**C–E**) Lysates from DEFs co-transfected with plasmids encoding HA-NS5 and Flag-TOLLIP (**C**), Flag-OPTN (**D**), or Flag-NBR1 (**E**), together with or without MYC-MARCH6, and treated with Baf A1 (10 µM), were subjected to co-IP assays using an anti-Flag antibody, followed by immunoblot analysis with the indicated antibodies. (**F–H**) Lysates from DEFs co-transfected with specific siRNA targeting MARCH6 and plasmids encoding Flag-NS5 and HA-TOLLIP (**F**), HA-OPTN (**G**), or HA-NBR1 (**H**) were subjected to co-IP assays using an anti-Flag antibody, followed by immunoblot analysis with the indicated antibodies. (**I–K**) Immunoblot analysis of lysates of DEFs co-transfected with HA-NS5 and shTOLLIP (**I**), shOPTN (**J**), or shNBR1 (**K**), together with or without Flag-MARCH6 for 24 h. (**L and M**) DEFs were transfected with shNegative or shTOLLIP, along with Flag-MARCH6 or an empty vector for 24 h and then infected with TMUV at an MOI of 0.1. E protein expression was analyzed by Western blotting (**L**), and viral titers were quantified using the TCID_50_ assay (**M**). (M) Data are presented as mean values ± standard error of the mean of three independent experiments (Student’s *t*-test; **, *P* < 0.01) ns, not significant.

Accumulating evidence indicates that selective autophagy relies on cargo receptors to identify specific substrates for lysosomal degradation (27). Given that MARCH6 lacks intrinsic cargo receptor function, we hypothesized that MARCH6 might bridge between NS5 and a cargo receptor for autophagic degradation. To systematically identify the receptor that mediates this process, we performed co-IP in DEFs and compared the binding partners of MARCH6 and NS5 with five evolutionarily conserved autophagy receptors: SQSTM1, OPTN, NBR1, TOLLIP, and BNIP3L. The results showed that MARCH6 showed robust associations with OPTN, NBR1, and TOLLIP (Fig. 6A), while NS5 interacted with OPTN, NBR1, TOLLIP, and BNIP3L (Fig. 6B). Further analysis revealed that MARCH6 specifically enhanced the interaction between NS5 and TOLLIP, but not between NS5 and NBR1 or OPTN (Fig. 6C through E). In loss-of-function experiments in which MARCH6 was knocked down using siRNA, the formation of the NS5-TOLLIP complex was impaired (Fig. 6F through H). These results suggest that MARCH6 specifically facilitates the interaction between NS5 and TOLLIP. To determine which cargo receptor is critical for MARCH6-mediated degradation of NS5, we used parallel shRNA knockdown to assess functional significance. As shown in Fig. 6I, depletion of TOLLIP significantly blocked MARCH6-driven NS5 degradation, whereas knockdown of OPTN/NBR1 maintained the efficiency of degradation (Fig. 6J and K). Remarkably, knockdown of TOLLIP not only stabilized NS5 but also reversed the antiviral activity of MARCH6, as quantified by Western blotting analysis of viral protein accumulation (Fig. 6L) and infectious titer measurements (Fig. 6M). Our data altogether suggest that TOLLIP functions as a cargo receptor in the MARCH6-mediated autophagic degradation of TMUV NS5.

### MARCH6 bridges TOLLIP and NS5 via its transmembrane domain

**Fig 7:**
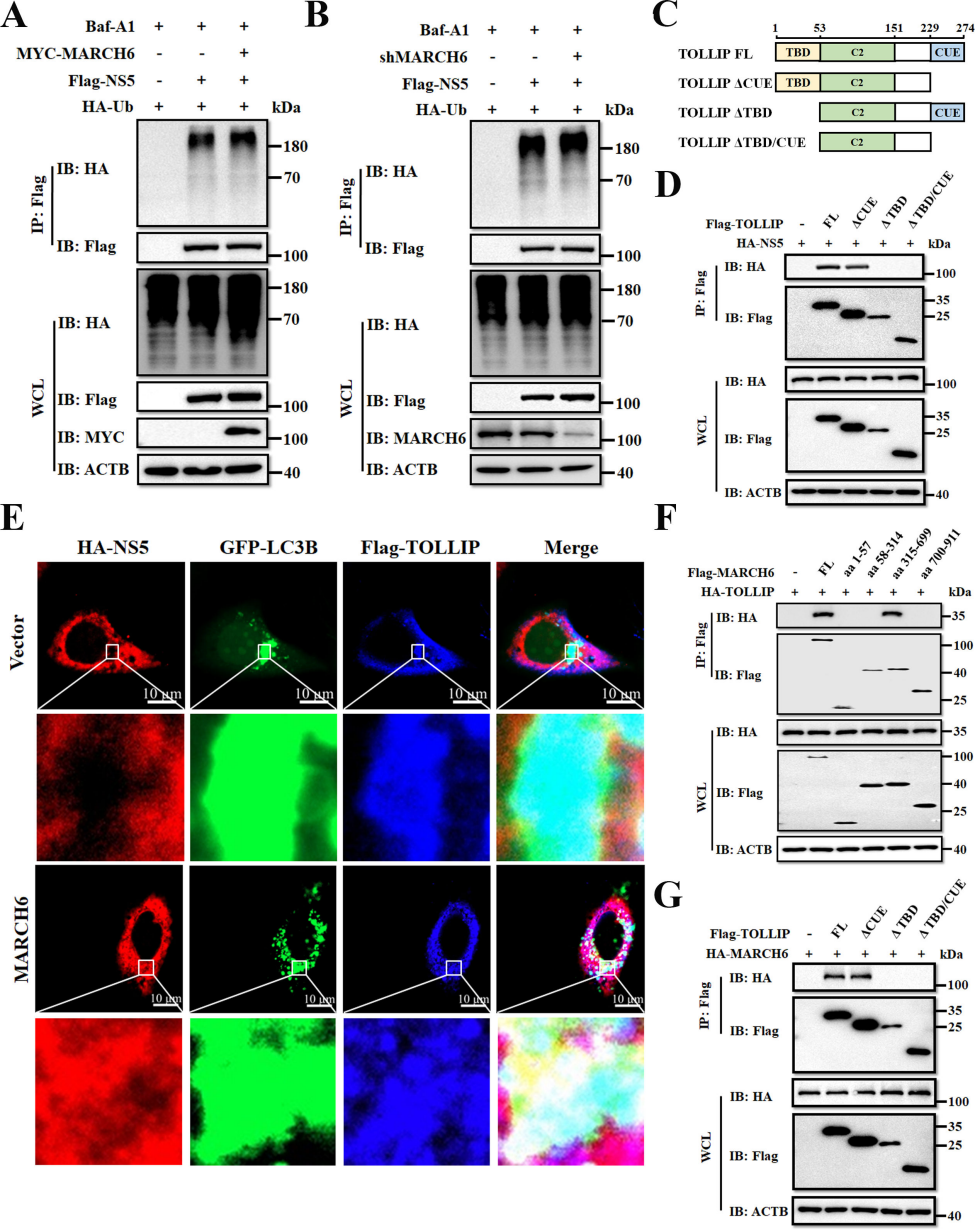
MARCH6 bridges TOLLIP and NS5 via its transmembrane domain. (**A and B**) Lysates of DEFs co-transfected with plasmids encoding HA-Ub and Flag-NS5, with or without MYC-MARCH6 (**A**) or shMARCH6 (**B**), and treated with Baf A1 (10 µM) were subjected to co-IP assays with an anti-Flag antibody, followed by immunoblot analysis with the indicated antibodies. (**C**) Schematic representation of TOLLIP domains. (**D**) Co-IP and immunoblot analysis of lysates from HEK-293T cells transfected with Flag-TOLLIP or its deletion mutants along with HA-NS5. (**E**) HeLa cells were co-transfected with HA-NS5, Flag-TOLLIP, and GFP-LC3B, together with MYC-MARCH6 or an empty vector, for 24 h. Cells were fixed with 4% paraformaldehyde and analyzed by confocal microscopy. (**F**) Co-IP and immunoblot analysis of lysates from HEK-293T cells transfected with Flag-MARCH6 or its truncated mutants, along with HA-TOLLIP. (**G**) Co-IP and immunoblot analysis with lysates from HEK-293T cells co-transfected with HA-MARCH6 and Flag-TOLLIP or its truncated mutants.

Previous structural studies have shown that the C-terminal CUE domain of TOLLIP recognizes ubiquitinated substrates for autophagic degradation (30, 43). To investigate whether MARCH6 functions as an E3 ligase that mediates ubiquitination of NS5, we examined the ubiquitination status of NS5 under MARCH6 modulation. Co-IP assays in DEFs showed no detectable changes in NS5 polyubiquitination upon MARCH6 overexpression (Fig. 7A) and unperturbed ubiquitination patterns after MARCH6 knockdown (Fig. 7B). Moreover, truncation of the ubiquitin-binding CUE domain of TOLLIP (ΔCUE mutant) retained its interaction with NS5 in DEFs (Fig. 7C and D), suggesting ubiquitin-independent recognition.

Because MARCH6 and TOLLIP mediate autophagic degradation of NS5 independently of NS5 ubiquitination, we hypothesized that MARCH6 might function as an adaptor protein that facilitates the interaction between NS5 and TOLLIP. Confocal microscopy showed that overexpression of MARCH6 enhanced the spatial arrangement of NS5/TOLLIP complexes in GFP-LC3B-marked autophagosomes (Fig. 7E), suggesting that MARCH6 recruits NS5 to TOLLIP for degradation. To identify the region of MARCH6 responsible for interacting with TOLLIP, we performed co-immunoprecipitation assays using Flag-tagged MARCH6 mutants. The results showed that the fragment spanning aa 315–699 mediates binding to TOLLIP (Fig. 7F). Notably, this region overlaps with the previously identified NS5-binding domain of MARCH6 (Fig. 4G), suggesting dual functionality in bridging both partners. Next, we determined which domain of TOLLIP is involved in the association with MARCH6. To do so, we co-transfected Flag-tagged TOLLIP deletion mutants with HA-MARCH6 into DEFs. Co-IP experiments showed that deletion of the Toll/interleukin-1 receptor binding domain (TBD) of TOLLIP abolished its interaction with MARCH6 (Fig. 7G), thereby pinpointing TBD as a critical interface. Taken together, these results suggest that MARCH6 facilitates the interaction between TOLLIP and NS5 via its TM domain, thereby promoting autophagic degradation of NS5.

## DISCUSSION

**Fig 8:**
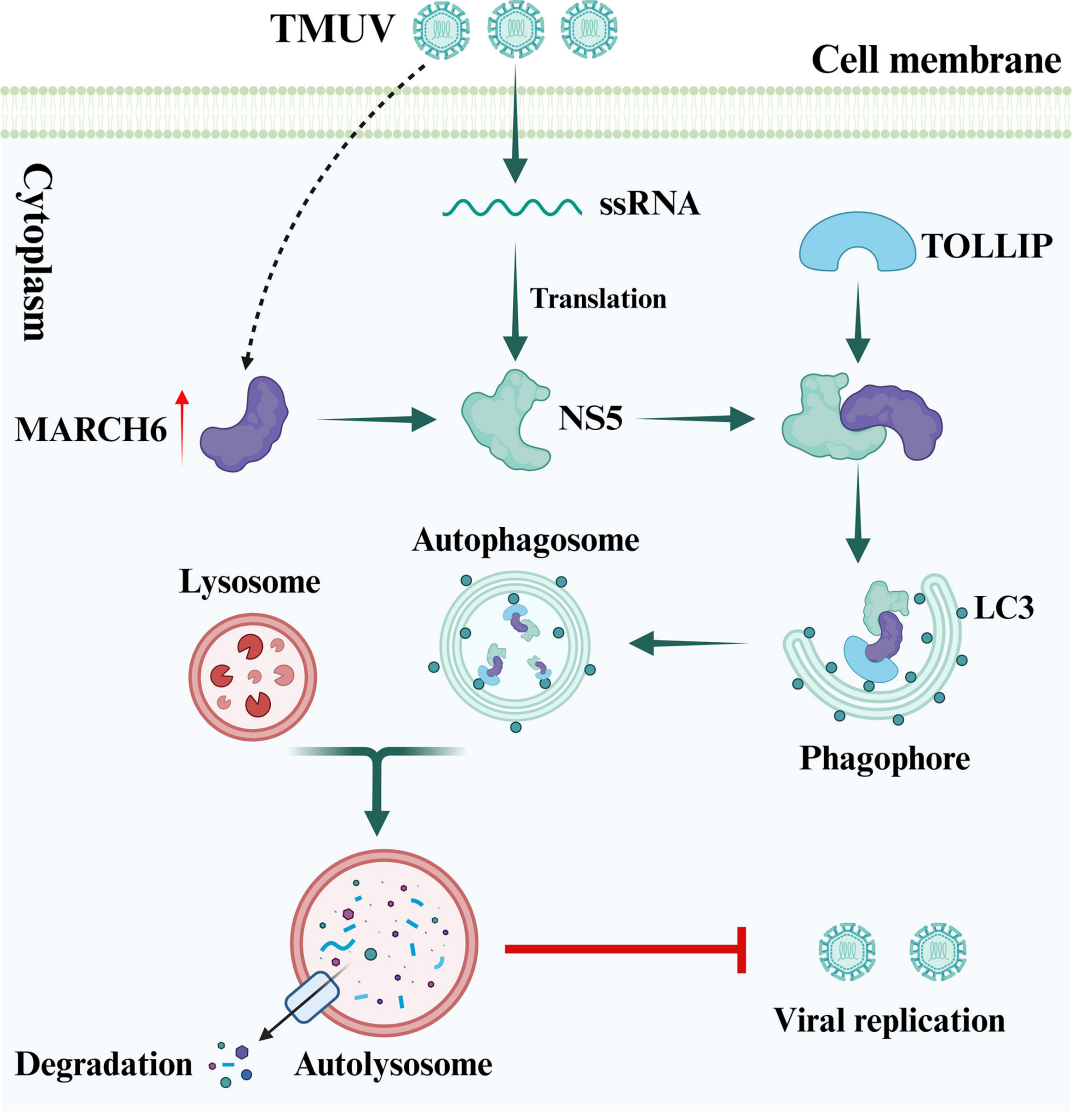
A proposed working model illustrating how the MARCH6-NS5-TOLLIP axis inhibits TMUV infection. Created in BioRender.

Autophagy plays a critical role in regulating viral replication (44, 45). Although several studies have shown that TMUV utilizes autophagy to facilitate its replication (46–48), the exact interplay between viral infection and autophagy has not been fully elucidated. In this study, we identified a novel antiviral mechanism in which the host protein MARCH6 inhibits TMUV replication through the selective autophagy pathway. Through our studies, we demonstrated that MARCH6 recruits the cargo receptor TOLLIP to promote autophagic degradation of the TMUV NS5 protein, thereby suppressing TMUV infection. This newly identified MARCH6-TOLLIP-NS5 axis represents the first evidence for the involvement of MARCH6 in the antiviral response (Fig. 8).

MARCH6, also known as TEB4 or RNF176, was originally identified for its role in degrading squalene monooxygenase and has been recognized as a key regulator of cholesterol biosynthesis and homeostasis (41, 49). While the role of MARCH6 in regulating viral replication remains unclear, other members of the MARCH family have been widely associated with the modulation of viral infections. For example, MARCH8 inhibits HIV-1 replication by downregulating viral envelope glycoproteins and retaining them intracellularly so that they cannot be incorporated into virions, thereby reducing the production of infectious viral particles (50). Additionally, MARCH5 plays a crucial role in inhibiting hepatitis B virus (HBV) replication by targeting the HBV X protein for proteasomal degradation (51). Our results showed that TMUV infection upregulates the expression of duck MARCH6 in DEFs and duck theca cells, which subsequently suppress TMUV replication. Knockdown of MARCH6 dramatically enhanced TMUV replication, demonstrating the role of MARCH6 as a host restriction factor against TMUV infection. Mechanistic studies revealed that MARCH6 specifically binds to the TMUV NS5 protein via its C-terminal transmembrane domain, resulting in the degradation of NS5 in DEFs. Interestingly, while the E3 ligase activity of MARCH6 is essential for metabolic regulation, our results indicate that it is not required for either inhibition of TMUV infection or NS5 degradation. This finding is consistent with previous studies by Yuan et al., who showed that TRIM25 inhibits rabies virus replication by destabilizing the viral phosphoprotein (P) independently of its E3 ubiquitin ligase activity (52). These observations suggest that MARCH6 facilitates the degradation of TMUV NS5 independently of MARCH6-mediated ubiquitination activity.

A growing body of evidence suggests that selective autophagy receptors play a crucial role in antiviral defense by directing viral proteins for degradation. For example, galectin-9 limits HBV replication by targeting the viral core protein (HBc) for selective autophagy via the autophagy receptor SQSTM1/p62 (53). Similarly, BST2/tetherin inhibits porcine epidemic diarrhea virus replication by promoting the autophagic degradation of the viral nucleocapsid (N) protein through CALCOCO2/NDP52 (54). Building on these established mechanisms, we identified duck TOLLIP as the key cargo receptor involved in MARCH6-mediated degradation of TMUV NS5. Our experimental data demonstrated that overexpression of MARCH6 enhanced the interaction between NS5 and TOLLIP in DEFs, whereas silencing of MARCH6 significantly reduced their association. Furthermore, depletion of TOLLIP impaired the MARCH6-mediated degradation of NS5 in DEFs. Previous studies suggest that TOLLIP normally recognizes ubiquitinated substrates via its C-terminal CUE domain and directs them for degradation in autophagosomes (30, 43). However, our results showed that TOLLIP interacts with TMUV NS5 independently of its CUE domain. Most notably, neither overexpression nor repression of MARCH6 altered the ubiquitination level of NS5, suggesting a mechanism independent of ubiquitination. In addition, we observed that MARCH6 enhanced the co-localization of NS5 and TOLLIP in specific aggregates that were positive for the autophagosome marker GFP-LC3B. These observations collectively demonstrate that MARCH6 enhances the interaction between TMUV NS5 and the selective autophagic receptor TOLLIP, thereby promoting the transport of NS5 to phagophores for degradation.

Flavivirus NS5 is essential for viral replication. Its C-terminal RdRp domain synthesizes new viral RNA genomes, and its N-terminal MTase domain generates the cap1 structure at the 5′-end of the newly synthesized viral genomes (55). In addition to viral replication, NS5 also plays a crucial role in suppressing interferon signaling and thus contributes to viral survival (15). Due to its essential role in genome replication and evasion of the immune system, cellular host factors promote the degradation of NS5 as part of antiviral responses. In particular, degradation of STAT2, together with NS5, may represent a host strategy to limit viral replication (56). Our work demonstrates that avian MARCH6 facilitates the degradation of TMUV NS5 via the cargo receptor TOLLIP, thereby inhibiting viral replication. An important question for future research is whether MARCH6 also promotes the degradation of NS5 of other flaviviruses and limits their infection. This study is the first to identify a host protein that targets a TMUV protein for autophagic degradation, providing new insights into avian antiviral defense mechanisms.

In summary, our study identifies avian MARCH6 as a novel host restriction factor against TMUV infection that mediates the autophagic degradation of the viral NS5 protein via the MARCH6-TOLLIP-NS5 axis. Our findings not only reveal the role of MARCH6 in the host antiviral response but also provide new insights into the intricate interplay between viral infection and selective autophagy in birds.

## MATERIALS AND METHODS

### Cells and viruses

DEFs (ATCC, CCL-141) were maintained in minimum essential medium (Gibco, C12571500BT) supplemented with 10% fetal bovine serum (FBS) (Gibco, 16000044). HEK-293T (human embryonic kidney epithelial cell line; ATCC, CRL-3216), BHK-21 (baby hamster kidney cells; ATCC, CCL-10), and HeLa cells (human cervical cancer cell line; ATCC, CCL-2) were grown in Dulbecco’s modified Eagle’s medium (Gibco, 12800082) supplemented with 10% FBS (Gibco, 16000044). All cells were inoculated in a 37°C humidified incubator with 5% CO_2_. The TMUV strain MC (GenBank number: KX452096) was isolated and stored in our laboratory (57).

### Isolation of duck theca cells

The isolation and culture of duck theca cells were performed as previously described (58). Briefly, follicles at the F4-F2 stage were harvested from the ovaries of 2-year-old healthy laying ducks, washed with ice-cold sterile phosphate-buffered saline (PBS) (HyClone, SH30256), and carefully dissected to remove blood vessels and connective tissue and isolate the theca layers. The isolated theca layers were minced and digested with 0.2% collagenase II at 37°C with constant shaking for 30 min. Ice-cold PBS was then added to complete the digestion process. The resulting theca cell suspension was filtered through a 200-mesh filter, centrifuged at 800 × *g* for 10 min at room temperature, and resuspended in M199 medium (HyClone, SH30253) supplemented with 20% FBS (Gibco, 16000044). The cells were cultured in a humidified incubator at 37°C and 5% CO_2_, and the medium was changed after 6 h of incubation.

### Antibodies and reagents

The following antibodies were used in this study: rabbit anti-ACTB (AC026), rabbit anti-TOLLIP (A21551), rabbit anti-BECN1 (A21191), rabbit anti-ATG5 (A19677), horseradish peroxidase (HRP)-conjugated goat anti-rabbit IgG (H + L) (AS014), and HRP-conjugated goat anti-mouse IgG (H + L) (AS003) from ABclonal Biotechnology; mouse anti-OPTN (sc-271549) from Santa Cruz Biotechnology; rabbit anti-NBR1 (DF12049) from Affinity Biosciences; rabbit anti-MAP1LC3B/LC3B (14600-1-AP) from Proteintech; rabbit anti-HA (561), rabbit anti-Flag (PM020), mouse anti-MYC (M192-3), mouse anti-HA (M180-3), and mouse anti-Flag (M185-3L) from MBL International Corporation; Dylight 405-conjugated anti-mouse IgG (35501BID), Alexa Fluor 488-conjugated anti-rabbit IgG (A21206), and Alexa Fluor 594-conjugated anti-rabbit IgG (A21207) from Thermo Fisher Scientific; mouse anti-TMUV E protein (clone 63D1E10) and mouse anti-duck MARCH6 (clone 5E1C7) were produced and stored in our laboratory; the anti-TMUV NS1 monoclonal antibody and anti-NS5 rabbit polyclonal antibody were kindly provided by Professor Shun Chen from Sichuan Agricultural University, Chengdu, China. Reagents used include EBSS (24010043, Gibco), Protein A/G Magnetic Beads (B23202, Selleck Chemicals), MG132 (C-2211-5MG, Sigma), 3-MA (S2767, Selleck Chemicals), CQ (PHR1258, Sigma-Aldrich), bafilomycin A1 (Baf A1) (S1413, Selleck Chemicals), CHX (S7418, Selleck Chemicals), and NH_4_Cl (ST2030, Beyotime Biotechnology).

### Plasmids and transfection

The full-length cDNAs encoding MARCH6, SQSTM1/p62, OPTN, NBR1, BNIP3L, and TOLLIP were amplified from DEFs and cloned into either the pCAGGS-Flag (MiaoLingBio, P1267) or pCAGGS-HA (MiaoLingBio, P0166) vectors. The TMUV protein-encoding genes were cloned into either the pCAGGS-HA or pCAGGS-Flag vector, as previously described (59). For knockdown of duck MARCH6, ATG5, BECN1, TOLLIP, OPTN, or NBR1 in DEFs, the shRNA oligonucleotides were annealed and cloned into the pGPU6/GFP vector (kindly provided by Professor Shun Chen from Sichuan Agricultural University, Chengdu, China). The primers are listed in Table S1. All plasmid constructs were verified by sequence analysis. These plasmids were transfected with JetPRIME (Polyplus-transfection, SA101000046) according to the manufacturer’s instructions.

### RNA interference

siRNAs targeting duck MARCH6, ATG5, and BECN1, along with an siNegative, were designed and synthesized by GenePharma (Shanghai, China). The sequences of these siRNAs are listed in Table S2. These siRNAs were transfected into DEFs using Lipofectamine 2000 reagent (Thermo Fisher Scientific, 11668019) according to the manufacturer’s instructions.

### RT-qPCR

Total RNA was extracted from the cells using TRIzol reagent (Vazyme, R401-01) according to the manufacturer’s instructions. For reverse transcription-qPCR, cDNA was synthesized using HiScript II 1st Strand cDNA Synthesis Kit (Vazyme, R232-01), and qPCR was performed using ChamQ Universal SYBR qPCR Master Mix (Vazyme, Q711). The expression levels of the indicated genes were normalized to the internal control GAPDH. All experiments were performed at least three times, and relative mRNA expression levels were quantified using the 2^−ΔΔCt^ method. The sequences of all primers used are listed in Table S1.

### Western blotting

Cell lysates were prepared with a lysis buffer containing 65 mM Tris-HCl, 4% sodium dodecyl sulfate, 3% DL-dithiothreitol, 40% glycerol, and protease inhibitor cocktail (Sigma, P8340). These lysates were boiled for 10 min in 5× SDS-PAGE sample loading buffer (Beyotime, P0015L), except for MARCH6 immunoblots, which were heated at 65°C for 15 min. Equal volumes of these lysates were then separated on 12% SDS-PAGE gels and transferred onto polyvinylidene fluoride membranes (Amersham Hybond P, 10600023). The membranes were blocked with 5% non-fat milk and incubated with the corresponding primary and secondary antibodies. Immunolabeled protein complexes were visualized using the WesternBright ECL kit (Advansta, K-12045-D50), and images were captured using the ECL detection system (Tanon-5200, Tanon).

### Co-immunoprecipitation

At 24 h post-transfection, cells were lysed using radioimmunoprecipitation assay (RIPA) buffer (Beyotime, P0013B) supplemented with a protease inhibitor cocktail (Sigma, P8340). The lysates were centrifuged at 12,000 × *g* for 15 min to remove cellular debris, and a portion of the supernatant was collected for total cell extract analysis. The remaining supernatant was incubated with magnetic beads conjugated with anti-Flag or anti-HA antibodies for immunoprecipitation. The beads were then washed five times with RIPA buffer, and the immunoprecipitated proteins were eluted with SDS loading buffer. Finally, the proteins were analyzed by a standard immunoblotting assay.

### Cell treatment

For protein degradation inhibition assays in DEFs, autolysosome- or lysosome-mediated protein degradation was inhibited with 3-MA (10 mM), CQ (10 µM), NH_4_Cl (20 mM), or Baf A1 (10 µM). Proteasome-mediated degradation was inhibited by MG132 (10 µM), while caspase-mediated degradation was blocked by Z-VAD-FMK (50 µM). Autophagy was induced with Torin1 (250 nM) or EBSS. Protein synthesis was inhibited with CHX (100 µg/mL).

### Indirect immunofluorescence assay

At 24 h post-transfection, HeLa cells were fixed with 4% paraformaldehyde for 15 min, permeabilized with 0.1% Triton X-100 (Sigma, T8787) for 15 min, and blocked with 5% bovine serum albumin for 1 h. The cells were then incubated with the primary antibody, diluted in PBS containing 0.5% Tween-20 for 1 h, followed by incubation with a fluorescently labeled secondary antibody for 1 h. Finally, cells were stained with 4′,6-diamidino-2-phenylindole (Solarbio, C0060) for 10 min. Fluorescence images were captured using a Zeiss LSM510 Meta confocal microscope (Carl Zeiss, Jena, Germany).

### TCID_50_ assay

The TCID_50_ assays were performed as described previously (60). Briefly, samples were serially diluted and inoculated into 96-well plates containing DEFs. After a 4 day incubation period, cells were analyzed under the microscope for cytopathic effects. Virus titers, expressed as TCID_50_/mL, were calculated using the Reed-Muench method.

### Statistical analysis

Data were analyzed using GraphPad Prism software. The significance of the differences was determined using an unpaired, two-tailed Student’s *t*-test. Data are presented as mean ± standard error of the mean. *P < 0.05 was considered statistically significant, and **P < 0.01 was considered highly significant.

## Data Availability

Data will be made available on reasonable request.

## SUPPLEMENTAL MATERIAL

Supplemental material jvi.00735-25-s0001.docx Fig. S1; Tables S1 and S2.

ASM does not own the copyrights to Supplemental Material that may be linked to, or accessed through, an article. The authors have granted ASM a non-exclusive, world-wide license to publish the Supplemental Material files. Please contact the corresponding author directly for reuse.
