# Supplementary material for: MARCH6 suppresses Tembusu virus replication by targeting viral NS5 protein for TOLLIP-mediated selective autophagic degradation
Source: J Virol. 2025 Jun 13;99(7):e00735-25. doi: 10.1128/jvi.00735-25 (PMC12282148; doi:10.1128/jvi.00735-25)
Supplement: Supplemental material — Fig. S1; Tables S1 and S2. [file jvi.00735-25-s0001.docx]

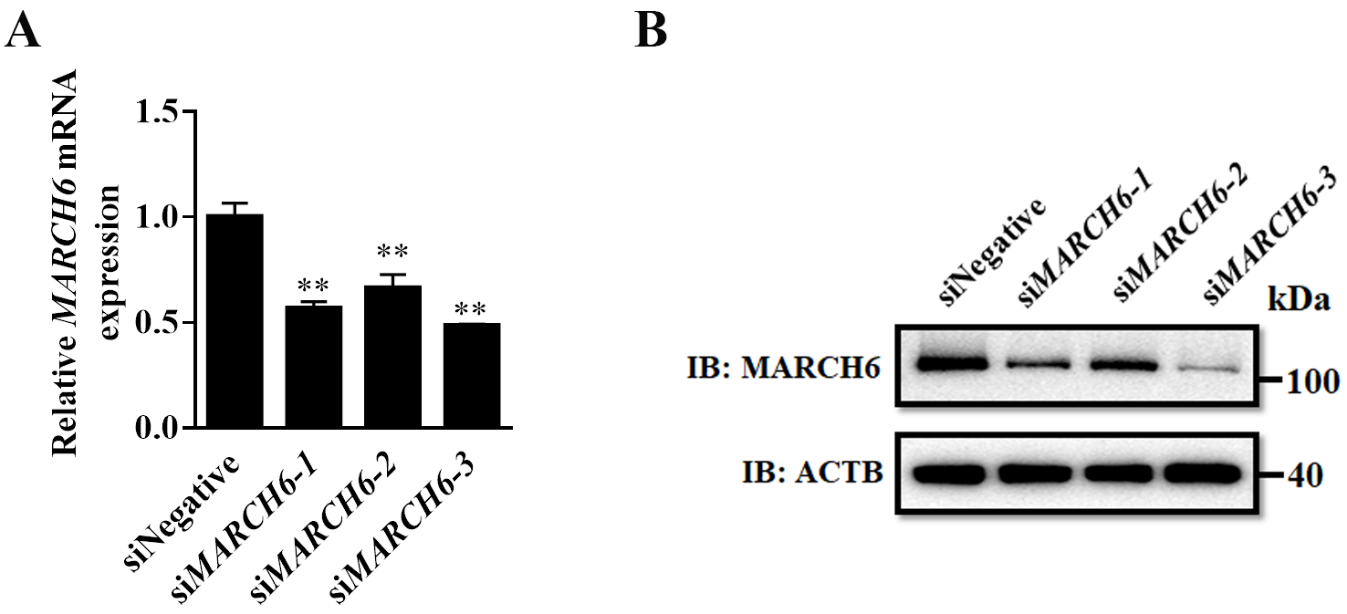


**Figure S1.** Gene silencing efficiency of MARCH6-targeting siRNA in DEFs. (A) RT-qPCR analysis of DEFs transfected with siNegative or siRNA targeting MARCH6 (siMARCH6). Data are represented as mean ± SEM of three independent experiments. **P*< 0.05 and ***P* < 0.01 (unpaired Student's *t*-test). (B) Immunoblot analysis of DEFs transfected with siNegative or siMARCH6.

**Table S1.** Primers for plasmid construction and RT-qPCR in this study.

| Primer | Sequence (5’-3’) |
| --- | --- |
| *MARCH6-F* | TATGAATTCATGGAGACCGCCGAGGAAGCAGATATATG |
| *MARCH6-R* | TATCTCGAGCTATTCTTGCGAAGACTGAGGTGGAGTTG |
| *MARCH6^C10A^-F* | TATGAATTCATGGAGACCGCCGAGGAAGCAGATATAGCTAGAGTCTGTC |
| *MARCH6^C10A^-R* | TATCTCGAGCTATTCTTGCGAAGACTGAGGTGGAGTTG |
| *MARCH6 RING-F* | TATGAATTCATGGAGACCGCCGAGGAAG |
| *MARCH6 RING-R* | GCCTCGAGTTACTTACATAATTCACAGTACTCTTTTCTG |
| *MARCH6 TM-F* | GCGAATTCATGCACAGATTTGCTTTCACACCAATTTATTC |
| *MARCH6 TM-R* | TATCTCGAGCTATTCTTGCGAAGACTGAGGTGGAGTTG |
| *TMUV NS5-F* | TATGAATTCGGAGGGGGGACTGGCAGAACTTTG |
| *TMUV NS5-R* | TATCTCGAGCAAGACACCTTCACTCCAGCTTTCAATG |
| *TMUV NS5 MTase-F* | TATGAATTCATGGGAGGGGGGACTGGCAGAACTTTG |
| *TMUV NS5 MTase-R* | TATCTCGAGCCTGGGTCCATTGCGGTTGGTTTTGTG |
| *TMUV NS5 RdRp-F* | GCGAATTCATGTATGAAGAAGATGTGGACTTGGGTTCAG |
| *TMUV NS5 RdRp-R* | TATCTCGAGCAAGACACCTTCACTCCAGCTTTCAATG |
| *TOLLIP-F* | TATGAATTCATGGCCACCACCGTCAGCAC |
| *TOLLIP-R* | GCCTCGAGCTATGATTCTTCAGCCATCTG |
| *TOLLIPΔCUE-F* | TATGAATTCATGGCCACCACCGTCAGCAC |
| *TOLLIPΔCUE-R* | TATCTCGAGCTACAGGTGCTGAGGGTTG |
| *TOLLIPΔTBD-F* | GCGAATTCATGAGACTCAGCATTACTGTG |
| *TOLLIPΔTBD-R* | GCCTCGAGCTATGATTCTTCAGCCATCTG |
| *TOLLIPΔTBD/CUE-F* | GCGAATTCATGAGACTCAGCATTACTGTG |
| *TOLLIPΔTBD/CUE-R* | TATCTCGAGCTACAGGTGCTGAGGGTTG |
| *qTMUV-E-F* | ACCATGGACAGGGTCATCAG |
| *qTMUV-E-R* | GGAGGGCTCCTTCTTGTGAT |
| *qMARCH6-F* | AATGCTTTGGAATGGGATCG |
| *qMARCH6-R* | CCAGGCCAACAATGGAGAAG |
| *qGAPDH-F* | CAAGGCTGAGAATGGGAAACTT |
| *qGAPDH-R*  *shATG5-F*  *shATG5-R*  *shBECN1-F*  *shBECN1-R*  *shNBR1-F*  *shNBR1-R*  *shOPTN-F*  *shOPTN-R*  *shTOLLIP-F*  *shTOLLIP-R*  *shMARCH6-F*  *shMARCH6-R* | GCATCTGCCCACTTGATGTT  AATTCGGCTGTATCAGGATGAGATAACCTCGAGGTTATCTCATCCTGAT  ACAGCTTTTTTG  GATCCAAAAAAGCTGTATCAGGATGAGATAACCTCGAGGTTATCTCAT  CCTGATACAGCCG  AATTCGGTCAGTTTGGCACGATCAATACTCGAGTATTGATCGTGCCAA  ACTGACTTTTTTG  GATCCAAAAAAGTCAGTTTGGCACGATCAATACTCGAGTATTGATCGT  GCCAAACTGACCG  AATTCGTGGACAACATTCAGATCAAATCTCGAGATTTGATCTGAATGT  TGTCCATTTTTTG  GATCCAAAAAAGGAGTACTGCAAGAGATTATTCTCGAGAATAATCTC  TTGCAGTACTCCCG  AATTCGTCACTGAGAACAATGAGTTAACTCGAGTTAACTCATTGTTCT  CAGTGATTTTTTG  GATCCAAAAAATCACTGAGAACAATGAGTTAACTCGAGTTAACTCATT  GTTCTCAGTGACG  AATTCGGACAAAGAAGGAATGATTAATCTCGAGATTAATCATTCCTTC  TTTGTCTTTTTTG  GATCCAAAAAAGACAAAGAAGGAATGATTAATCTCGAGATTAATCAT  TCCTTCTTTGTCCG  AATTCGTGTGTGTACTGGCAGTATTAACTCGAGTTAATACTGCCAGTAC  ACACATTTTTTG  GATCCAAAAAATGTGTGTACTGGCAGTATTAACTCGAGTTAATACTGC  CAGTACACACACG |

**Table S2.** The sequences of siRNAs used in the study.

| siRNA | Sequences (5' to 3') |
| --- | --- |
| *siMARCH6-1*  *siMARCH6-2*  *siMARCH6-3*  *siBECN1*  *siATG5*  *siNegative control* | 5' GGAAGUGUCUGGAAGGCAUTT 3'  5' AUGCCUUCCAGACACUUCCTT 3'  5' CCAGUGGAACGGGAUCAAATT 3'  5' UUUGAUCCCGUUCCACUGGTT 3'  5' GCAGUUCGUCUGCAAAUAUTT 3'  5' AUAUUUGCAGACGAACUGCTT 3'  5' GCUCAGUACCAGAAGGAAUTT 3'  5' AUUCCUUCUGGUACUGAGCTT 3'  5' GGAUGUGAUUGAAGCUCAUTT 3'  5' AUGAGCUUCAAUCACAUCCTT 3'  5' UUCUCCGAACGUGUCACGUTT 3'  5' ACGUGACACGUUCGGAGAATT 3' |
